# PlaqueViT: a vision transformer model for fully automatic vessel and plaque segmentation in coronary computed tomography angiography

**DOI:** 10.1007/s00330-025-11410-w

**Published:** 2025-02-05

**Authors:** Jennifer Alvén, Richard Petersen, David Hagerman, Mårten Sandstedt, Pieter Kitslaar, Göran Bergström, Erika Fagman, Ola Hjelmgren

**Affiliations:** 1 Department of Electrical Engineering, Chalmers University of Technology, Gothenburg, Sweden; 2 Department of Radiology in Linköping, Linköping University, Linköping, Sweden; 3 Department of Health, Medicine and Caring Sciences and Center for Medical Image Science and Visualization (CMIV), Linköping University, Linköping, Sweden; 4 Medis Medical Imaging, Leiden, The Netherlands; 5 Department of Molecular and Clinical Medicine, Institute of Medicine, University of Gothenburg, Gothenburg, Sweden; 6 Department of Clinical Physiology, Queen Silvia Children’s Hospital, Sahlgrenska University Hospital, Region Västra Götaland, Gothenburg, Sweden; 7 Department of Radiology, Institute of Clinical Sciences, University of Gothenburg, Gothenburg, Sweden; 8 Department of Radiology, Queen Silvia Children’s Hospital, Sahlgrenska University Hospital, Region Västra Götaland, Gothenburg, Sweden; 9 Pediatric Heart Centre, Queen Silvia Children’s Hospital, Sahlgrenska University Hospital, Region Västra Götaland, Gothenburg, Sweden

**Keywords:** Coronary artery disease, Computed tomography angiography, Radiographic image interpretation, Computer-assisted

## Abstract

**Objectives:**

To develop and evaluate a deep learning model for segmentation of the coronary artery vessels and coronary plaques in coronary computed tomography angiography (CCTA).

**Materials and methods:**

CCTA image data from the Swedish CardioPulmonary BioImage Study (SCAPIS) was used for model development (*n* = 463 subjects) and testing (*n* = 123) and for an interobserver study (*n* = 65). A dataset from Linköping University Hospital (*n* = 28) was used for external validation. The model’s ability to detect coronary artery disease (CAD) was tested in a separate SCAPIS dataset (*n* = 684). A deep ensemble (k = 6) of a customized 3D vision transformer model was used for voxelwise classification. The Dice coefficient, the average surface distance, Pearson’s correlation coefficient, analysis of segmented volumes by intraclass correlation coefficient (ICC), and agreement (sensitivity and specificity) were used to analyze model performance.

**Results:**

PlaqueViT segmented coronary plaques with a Dice coefficient = 0.55, an average surface distance = 0.98 mm and ICC = 0.93 versus an expert reader. In the interobserver study, PlaqueViT performed as well as the expert reader (Dice coefficient = 0.51 and 0.50, average surface distance = 1.31 and 1.15 mm, ICC = 0.97 and 0.98, respectively). PlaqueViT achieved 88% agreement (sensitivity 97%, specificity 76%) in detecting any coronary plaque in the test dataset (*n* = 123) and 89% agreement (sensitivity 95%, specificity 83%) in the CAD detection dataset (*n* = 684).

**Conclusion:**

We developed a deep learning model for fully automatic plaque detection and segmentation that identifies and delineates coronary plaques and the arterial lumen with similar performance as an experienced reader.

**Key Points:**

***Question*** *A tool for fully automatic and voxelwise segmentation of coronary plaques in coronary CTA (CCTA) is important for both clinical and research usage of the CCTA examination.*

***Findings*** *Segmentation of coronary artery plaques by PlaqueViT was comparable to an expert reader’s performance.*

***Clinical relevance*** *This novel, fully automatic deep learning model for voxelwise segmentation of coronary plaques in CCTA is highly relevant for large population studies such as the Swedish CardioPulmonary BioImage Study.*

**Graphical Abstract:**

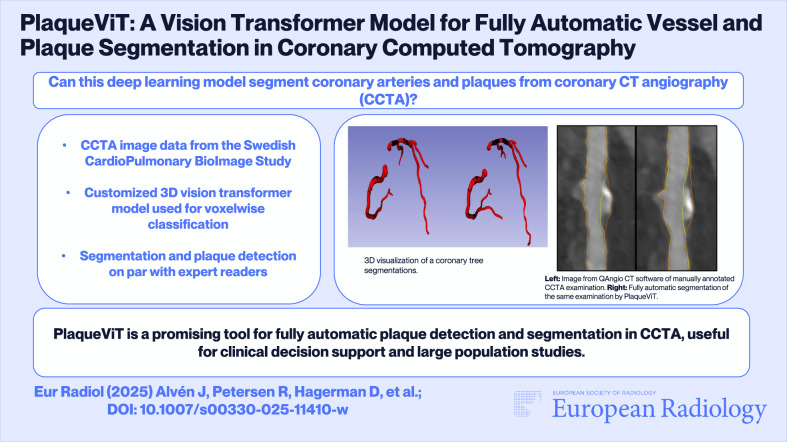

## Introduction

Coronary artery disease (CAD) is the predominant cause of morbidity and mortality in the Western world [1], and coronary computed tomography angiography (CCTA) is becoming the primary tool for its diagnosis and assessment [2]. Besides the degree of stenosis, CCTA can reveal features of coronary plaques, such as their size, shape, and composition [2]. Thus, by providing information beyond the traditional factors used to assess CAD risk, such as degree of stenosis, risk scores, and coronary artery calcifications, CCTA has the potential to enable more precise identification of persons at elevated risk of coronary events [3].

In several studies, plaque features identified by CCTA correlated with coronary events. In the SCOT-HEART trial, low-attenuation plaque volume was the best predictor of myocardial infarction in patients with chest pain [4]. In the ROMICAT 1 and 2 trials, positive remodeling, spotty calcifications, low-attenuation plaque volume, and stenosis length predicted acute coronary syndrome and remained a significant predictor after adjusting for stenosis and clinical risk assessment (age, gender, number of cardiovascular risk factors) [5–7]. However, data on risk prediction by CCTA plaque analysis is lacking in populations with asymptomatic coronary artery atherosclerosis. In the Swedish CardioPulmonary BioImage Study (SCAPIS) [8], designed to gather data for risk prediction in the general population, 30,154 participants aged 50–64 years were randomly recruited. Among those who underwent examinations including successful, high-quality CCTA (*n* = 25,182), 42.1% had subclinical atherosclerosis [9]. However, CCTA has not yet been used for detailed plaque analysis in SCAPIS, as the assessment is a time-consuming manual process that requires an experienced radiologist.

Previous studies of plaque composition [4–7] have used semi-automated methods to quantify the plaque burden. Deep learning methods have the potential to improve the speed and diagnostic performance of CCTA image analysis by providing automatic and objective results. A deep learning model would be invaluable both for the analysis of data from studies such as SCAPIS and for the clinical assessment of CCTA. Indeed, several attempts have been made to automate the detection and characterization of coronary plaques by voxelwise segmentation of CCTA [10–13]. For example, 3D convolutional neural networks [11, 13] and 2D convolutional long-short term memory networks [10] have been used to predict voxelwise plaque segmentations. However, these methods required preprocessing steps such as manual or semi-automatic centerline extraction and multiplanar reformation. In another study, a coronary artery and plaque segmentation method without the need for manual or semi-automatic centerline extraction is proposed; however, no evaluation of the voxelwise plaque segmentations is reported [12].

In this study, we sought to develop a fully automatic deep learning method for CCTA data analysis that was capable of coronary artery plaque segmentation without manual pre- or postprocessing, and that performed as well as an expert reader in CAD detection and plaque segmentation. This model will be referred to as PlaqueViT.

## Materials and methods

This study was approved by the ethical review boards in Gothenburg (570-18) and Linköping (2022-0541-01). SCAPIS was approved as a multicenter study by the ethical review board in Umeå (2010-228-31M). All participants gave written informed consent.

### Study population

**Fig. 1:**
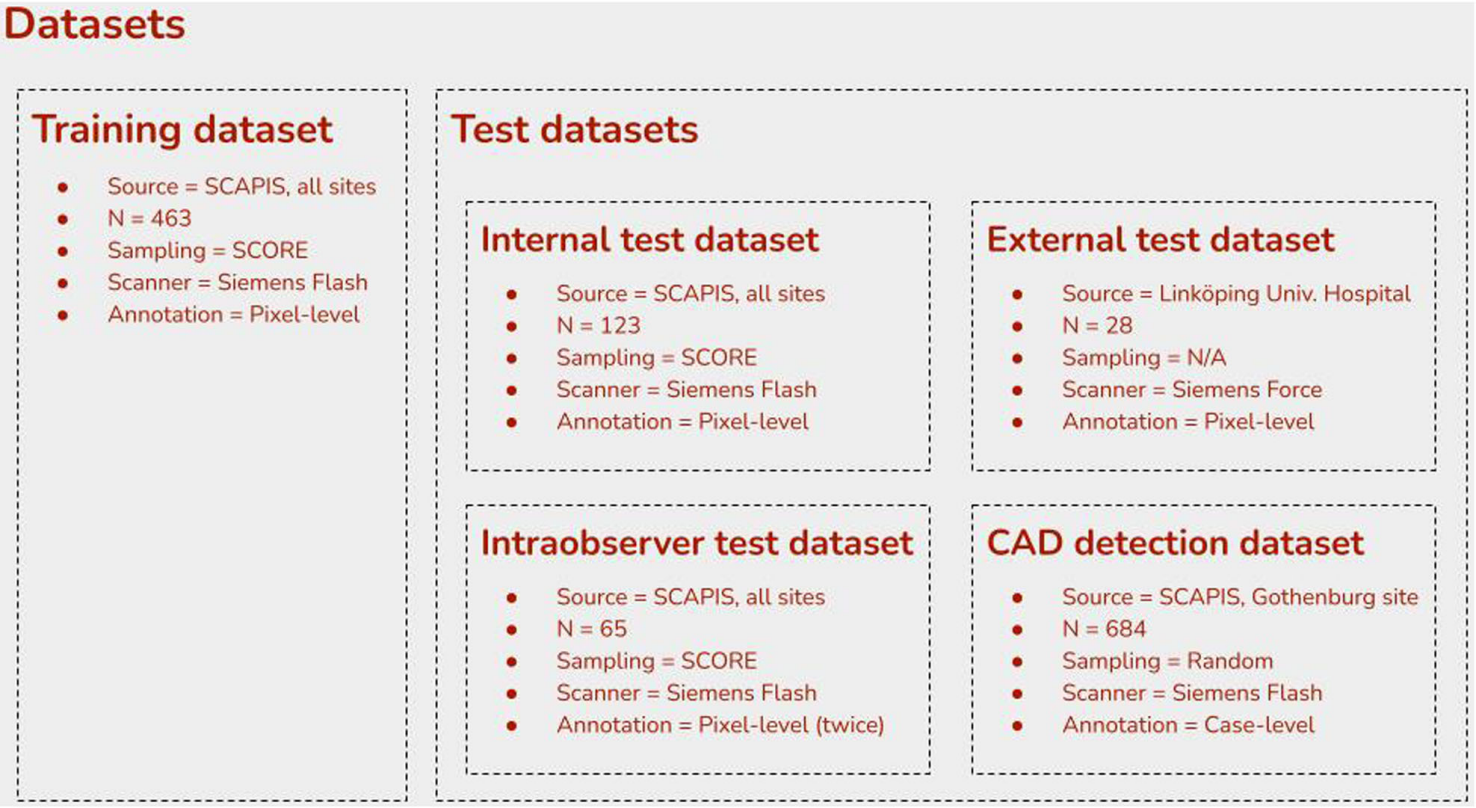
Overview of the datasets

Five retrospective CCTA datasets were used to train, validate, and test the model, (Fig. 1). No cases are included in more than one of the five different datasets. The training dataset and the internal test dataset were sampled from the SCAPIS cohort [8]. The criteria for inclusion in these datasets were (1) at least one coronary plaque detected by visual assessment in the SCAPIS, (2) a complete CCTA examination (tube voltage 100 kV), and (3) availability of the data needed to calculate risk with the Systematic COronary Risk Evaluation (SCORE) system (age, sex, smoking, systolic blood pressure, and total cholesterol). The exclusion criteria were (1) a previous myocardial infarction, stroke, or cardiac procedure (coronary artery bypass grafting or percutaneous coronary intervention) and (2) non-diagnostic examination due to low image quality. Exclusion due to low image quality was performed by the senior reader at an initial image quality check, and only non-diagnostic cases where image quality was too poor to delineate plaques in all proximal segments were excluded. In total, 7% of the CCTA scans were excluded. Equal numbers of eligible participants were randomized from each stratum of SCORE (0–1%, 2%, 3%, 4%, 5%, and ≥ 6%).

The intraobserver test dataset, designed to compare intraobserver variability, consisted of 36 subjects with CAD and 29 healthy subjects who met the eligibility criteria for the training and internal test datasets described above except that the healthy subjects had no plaques. The data sampling of the training, internal test, and intraobserver test datasets is described elsewhere [14]. The external test dataset [15] included 28 patients at low to intermediate risk for CAD who were referred for clinical CCTA at Linköping University Hospital.

The CAD detection dataset, designed to test the ability of the model to detect CAD, consisted of 684 cases, selected at random, that were not included in any of the other test datasets and that met the following inclusion criteria: (1) examination at the Gothenburg SCAPIS site, (2) a complete CCTA examination (tube voltage 100 kV), and (3) sufficient data from the primary radiological assessment in SCAPIS to classify the cases as healthy or as CAD positive. This CAD detection dataset did not include vessel or plaque contouring ground truth annotations, only case-level CAD status reports.

### Image acquisition

The computed tomography (CT) protocol in SCAPIS is described in detail elsewhere [8]. Briefly, CT was done on dedicated dual-source CT scanners (Somatom Definition Flash, Siemens Medical Solution). The examination included a non-contrast CT scan for calcium scoring and a CCTA scan with iodine contrast enhancement (Omnipaque, GE Healthcare, 350 mgI/mL) [16].

In the external validation dataset, all patients were examined on a dual-source CT scanner (Somatom Force, Siemens). High-pitch spiral CCTA scans were acquired prospectively if the patient’s heart rate was regular and below 65 beats/min. Otherwise, a retrospectively gated spiral scan was used. Image acquisition in subjects in the external validation dataset is described elsewhere [15]. The image preprocessing steps are described in the supplement.

### Image analysis

#### Expert CCTA annotations

Voxel-level annotations representing the ground truth were generated for the training dataset, the internal test dataset, the intraobserver test dataset and the external test dataset*,* with all annotations carried out in our core lab as previously described [14]. Plaques were analyzed with dedicated workstations and semi-automated plaque analysis software (Medis Suite CT, version 3.1.16.8, and QAngio CT RE, version 3.1.4.2, respectively; Medis Medical Imaging Systems). The annotation included contours of the coronary vessel wall and lumen, and all plaques were manually marked. All CCTA scans were analyzed by one of the four primary readers. The primary readers were experienced radiographers who had undergone specialized training in coronary plaque analysis using the Medis QAngio CT software. The training procedure has been previously described [14]. The annotations were subsequently reviewed and adjusted if necessary, by the senior expert reader (E.F., a senior radiologist). This workflow allowed a large volume of CCTA examinations to be processed by one single expert. CCTA scans with overall image quality deemed too poor for plaque analysis due to high noise levels or motion artifacts were excluded by the expert reader.

#### Intraobserver variability analysis

Two copies were made of each CCTA examination in the intraobserver test dataset and assigned new identification codes. Examinations were shuffled, and one copy from each pair was presented in a random order to two primary readers (Observers 1 and 2). All primary analyses were then sent to the senior reader in random order. The senior reader reviewed the annotations and made changes if deemed necessary. The resulting annotations from Observer 1 and 2 were used to assess intraobserver variability.

#### External test dataset analysis

All examinations in the external test dataset were separately analyzed and annotated, in blinded fashion, by two senior readers (E.F. and M.S.). Our core lab procedure was used for analysis and annotation [14].

#### Matched plaque analysis

To further evaluate the PlaqueViT model, all scans in the interobserver and external test datasets were examined visually, and plaques that overlapped spatially in the paired readings were labeled and analyzed pairwise. This visual confirmation ensured that the same plaque was quantified by Observer 1, Observer 2, and the PlaqueViT model.

#### CAD detection dataset analysis

All CCTA scans in the SCAPIS study were scored for CAD by trained radiologists or cardiologists, as described in [9]; the coronary segments were visually examined for the presence of plaques, and each segment was classified as follows: no atherosclerosis, 1–49% stenosis, or > 50% stenosis. In the present study, data was retrieved from the SCAPIS database regarding the presence of atherosclerosis (yes/no) in individual cases, which was used as ground truth. No voxelwise annotations were generated for the CAD detection dataset.

### Deep learning model

A one-step approach was used to directly segment the coronary artery lumen and plaques in the reconstructed 3D CCTA scan. PlaqueViT employs a customized version of the nnFormer model [17] which has a 3D vision transformer architecture that incorporates local multi-head self-attention and follows a U-net-like architecture with an encoder and a decoder, including an initial embedding layer and interleaved transformer and convolutional blocks. The initial embedding layer consists of 3D convolutional layers for accurate spatial encoding and extraction of high-resolution, low-level features. The transformer blocks use local multi-head self-attention and shifted window partitioning to incorporate long-term dependencies into high-level features, and the convolutional blocks model object concepts from high-level features at multiple scales. PlaqueViT’s model configurations differ from the original design. The number of local multi-head self-attention heads was set to 3, 6, 12, and 24 and the number of embedding dimensions to 96. Local multi-head attention was applied to a window of 4 × 4 × 4 voxels throughout all stages. Instead of deep supervision, stochastic depth, and skip-attention as proposed in the original work, standard residual connections between the encoder and decoder are used. See the supplement for more details on the architectural changes and model selection in an ablation study.

Model training used data augmentation of 90-degree rotations and mirroring along all dimensions. The model was trained as a deep ensemble with k = 6 ensemble members, each trained and validated on a separate data partition using k-fold with k = 6 on 463 images. During inference, the argmax of the average softmax over the ensemble is used as final prediction [18]. Validation was done every 1000 iterations, and the model with the best validation plaque Dice index was selected. An Nvidia DGX-2 supercomputer with four Nvidia Tesla V100 SXM3 32 GB HBM2 accelerators was used for training and validation.

### Statistical analysis

The performance of the model in lumen and plaque segmentations was assessed with the Dice coefficient and average surface distance. Plaque volumes were assessed with Pearson correlation, ICC, and limits of agreement, and mean absolute percentage error. Sensitivity, specificity, positive and negative predictive values were used to assess the performance of the model in detecting CAD, defined as plaque > 2 mm^3^. Statistical analysis was performed with the XLMiner Analysis ToolPak.

## Results

The characteristics of the subjects are summarized in Table 1. The training dataset and the internal test dataset consisted of 586 fully annotated cases, which were randomly assigned 80% to the former (*n* = 463) and 20% to the latter (*n* = 123). Validation was performed using k-fold cross-validation within the training dataset. The intraobserver test dataset consisted of 65 cases, of which 36 had at least one coronary plaque. The external test dataset included 28 cases, of which 17 had at least one coronary plaque. The CAD detection dataset consisted of 684 cases (341 subjects with CAD and 343 subjects without CAD).

**Table 1:** Characteristics of included subjects

Variable	Training dataset (*n* = 463)	Internal test dataset (*n* = 123)	Intraobserver test dataset (*n* = 65)	External test dataset (*n* = 28)	CAD detection dataset (*n* = 684)
Mean age, years (range)	61.5 (57.8–63.6)	61.2 (58.9–63.6)	61.1 (57.8–63.5)	60.3 (50–71)	57.6 (53.9–63.4)
Female sex, % (*n*)	18% (82)	19% (23)	14% (9)	54% (15)	49% (334)
Weight, kg	83 (74.2–91.0)	81.9 (75.7–94)	81.2 (71.7–90.0)	80 (74–92)	80.5 (69.6–90.9)
BMI, kg/cm^2^	26.6 (24.2–29.0)	26.9 (24.5–29)	26.3 (23.5–29.3)	27.8 (22.4–41.5)	26.4 (24.1–29.4)
Total cholesterol, mmol/L	5.7 (5.1–6.4)	5.7 (4.9–6.5)	5.70 (4.95–6.50)	*	5.50 (4.80–6.20)
Systolic blood pressure, mm Hg (range)	136 (123–150)	136 (124–152)	131 (121–146.5)	150 (128–162)	122 (111–133)
Diastolic blood pressure, mm Hg (range)	81 (74–89)	83 (72–90)	80 (72.5–86.50)	84 (77–91)	73 (66–82)
Current smoker, % (*n*)	32% (17)	33% (40)	39% (25)	3.6% (1)	12.9% (88)
Radiation dose, dose length product (mGy*cm)	85 (69–105)	85 (67–106)	83 (68–105.5)	156 (6–273)	79 (62–103)
Calcium score > 0%	83% (383)	86% (106)	52.3% (34)	46% (13)	46% (317)
Calcium score, Agatston units (range) For subjects with CACS > 0	70 (16–208)	60.5 (8–212.5)	34 (8–173)	331 (2–1140)	39 (9–138)
SCORE (range)	3.25 (1.7–4.86)	3.43 (1.96–5.00)	3.07 (1.83–4.95)	*	1.08 (0.59–1.80)

Data presented as median (IQR) or percentage (counts)

* External test dataset lacks information on cholesterol and SCORE

### Inference

**Fig. 2:**
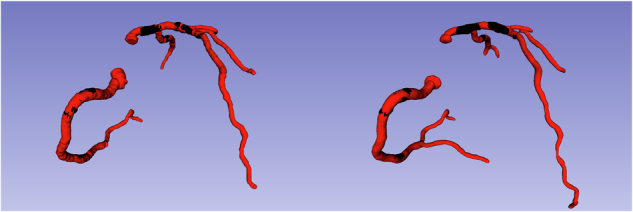
3D visualization of coronary tree segmentations. Left: A 3D visualization of a manually segmented coronary tree by Observer 1. Right: Same examination segmented by PlaqueViT. The vessel lumen voxels are shown in red. Coronary plaque voxels are shown in black

**Fig. 3:**
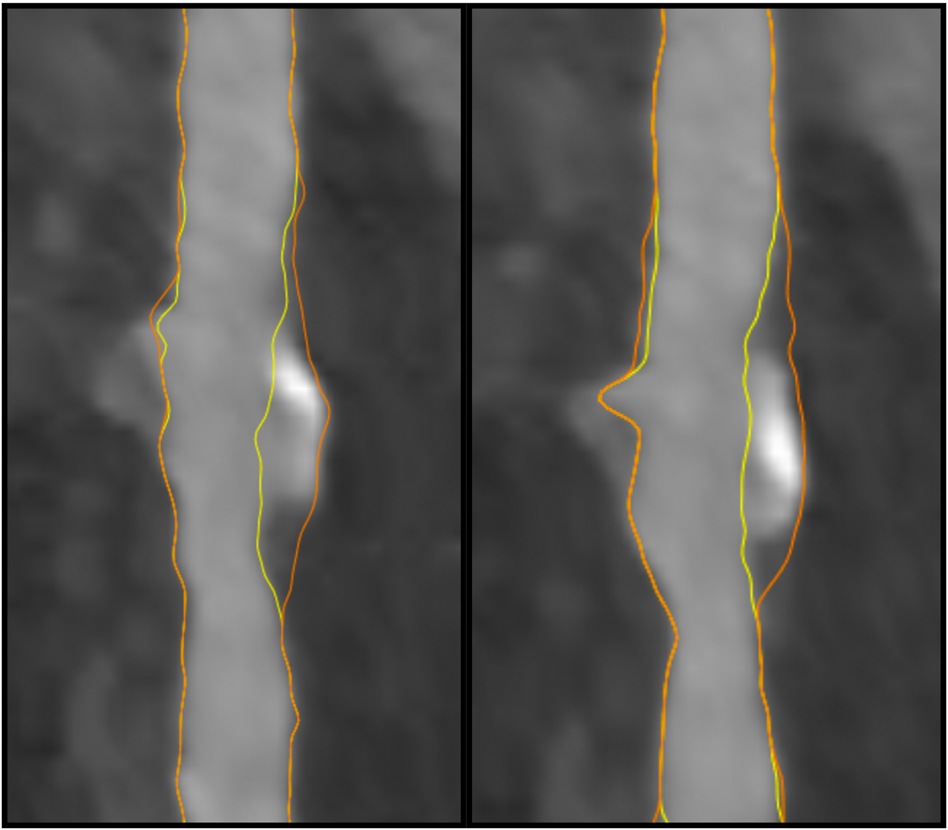
Example of coronary plaque. Left: Image from QAngio CT software of manually annotated CCTA examination showing a plaque in left anterior descending coronary artery. Yellow line, segmented vessel lumen. Orange line, outer contour of the vessel wall/outer wall of coronary plaque. Right: Fully automatic segmentation of the same examination by PlaqueViT (segmentation imported into QAngio CT software for visualization)

PlaqueViT fully segmented lumen and plaque voxels in the CCTA scans without any manual intervention. One example of our automatic plaque segmentation is visualized as a 3D volume in Fig. 2, and a detailed example of plaque annotation and corresponding model segmentation is shown in Fig. 3. PlaqueViT analysis was performed using a Nvidia Quadro RTX 8000 graphics card with a mean inference time of 25 s per case.

### Model performance in plaque segmentation

**Fig. 4:**
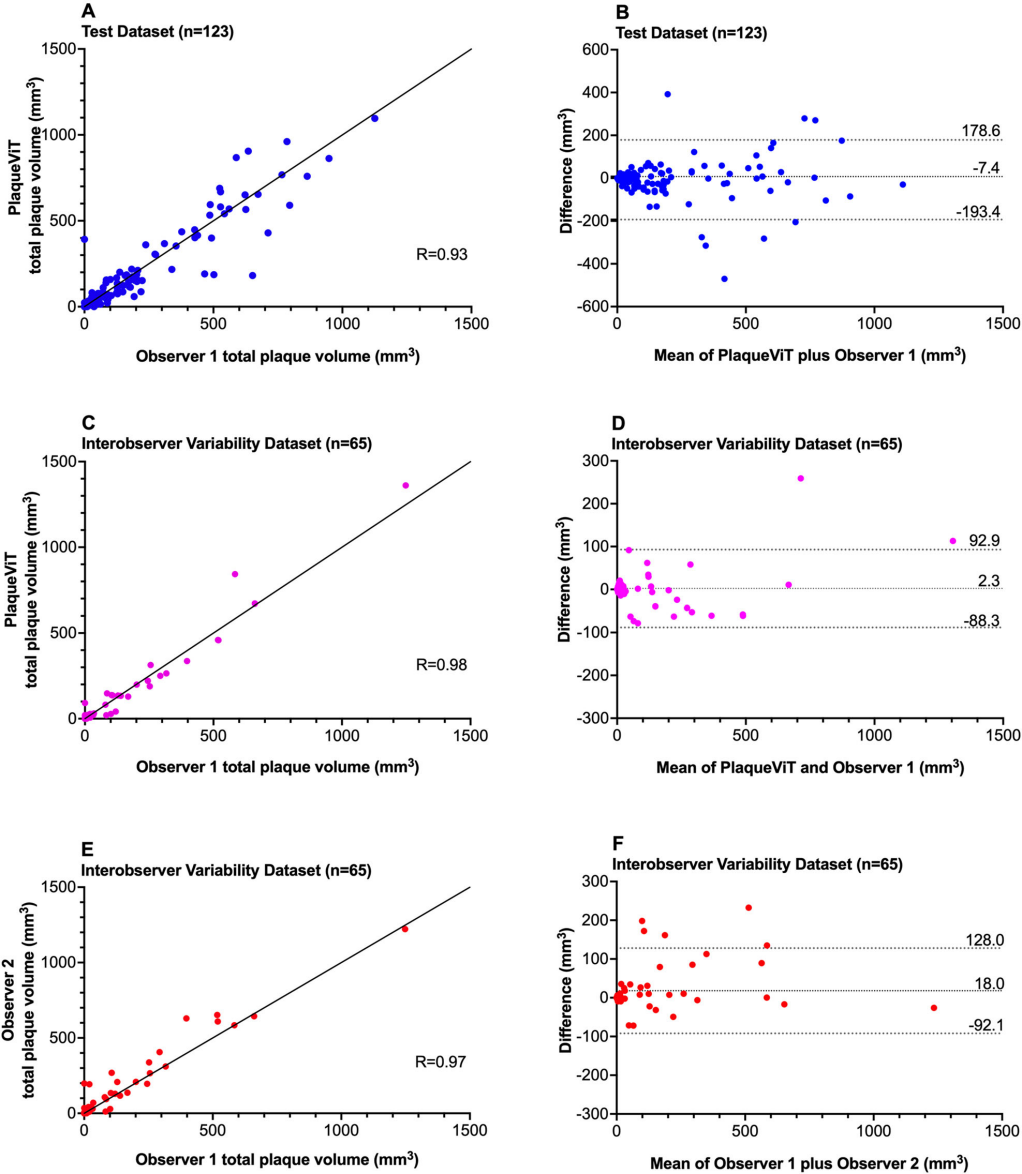
Total plaque volume measured by Observer 1 versus PlaqueViT and Observer 2. Correlation (**A**) and Bland-Altman (**B**) plots between PlaqueViT and Observer 1 in the test dataset. Correlation (**C**) and Bland-Altman (**D**) plots between PlaqueViT and Observer 1 in the interobserver variability dataset. Correlation (**E**) and Bland-Altman (**F**) plots between Observer 1 and Observer 2 in the interobserver variability dataset

The agreement between the segmented plaque volumes by PlaqueViT and the expert reader was excellent in the internal test dataset (ICC = 0.93) and the intraobserver test dataset (ICC = 0.98). The PlaqueViT model and expert annotations of total plaque volume in the internal test dataset and the intraobserver test dataset were strongly correlated (*r* = 0.93 and 0.98, respectively, *p* < 0.001) (Fig. 4).

In the intraobserver test dataset, the mean difference in total plaque volume between PlaqueViT and the expert reader was 2.3 mm^3^ (95% limits of agreement −88.3 to 92.9); the difference between Observers 1 and 2 was 8.3 mm^3^ (95% limits of agreement −92.0 to 128.0), as shown by Bland-Altman analysis. The mean Dice coefficient for segmentation of all plaques between PlaqueViT and expert was 0.55 in the internal test dataset and 0.51 in the intraobserver test dataset. This was slightly better than the mean Dice coefficient for Observer 1 versus Observer 2 (0.50). The median average surface distance between PlaqueViT segmentation and expert annotation was 0.98 mm in the internal test dataset and 1.31 mm in the intraobserver test dataset. The median average surface distance was 1.15 mm for Observer 1 versus Observer 2. Similarity metrics are shown in Table 2.

**Table 2:** PlaqueViT model performance in plaque segmentation

	Internal test dataset (*n* = 123)	Intraobserver test dataset (*n* = 65)	
Measurement	PlaqueViT versus Observer 1	PlaqueViT versus Observer 1	Observer 1 versus Observer 2
Segmentation of coronary plaques			
Dice coefficient	0.55 (0.21)	0.51 (0.20)	0.50 (0.24)
Median average surface distance (mm)	0.98	1.31	1.15
Total plaque volume (mm^3^)			
Mean percentage error	−9.7% (58.9)	17.4% (77.0)	12.1% (73.1)
Mean absolute percentage error	38.3% (45.5)	43.6% (66.8)	39.9% (62.3)
Correlation	0.93, *p* < 0.001	0.98, *p* < 0.001	0.97, *p* < 0.001
ICC	0.93, *p* < 0.001	0.98, *p* < 0.001	0.96, *p* < 0.001
Limits of agreement	−7.4 (−193.4 to 178.6)	2.3 (−88.3 to 92.9)	18.0 (−92.1 to 128.0)
Segmentation of coronary lumen			
Dice coefficient	0.81 (0.11)	0.85 (0.07)	0.91 (0.06)
Median average surface distance (mm)	1.65	0.71	0.45
Total lumen volume (mm^3^)			
Mean percentage error	8.4% (24.6)	−1.7% (15.6)	−5.7% (11.8)
Mean absolute percentage error	16.1% (20.7)	8.8% (13.0)	7.9% (10.3)
Correlation	0.82, *p* < 0.001	0.93, *p* < 0.001	0.96, *p* < 0.001
ICC	0.80, *p* < 0.001	0.92, *p* < 0.001	0.95, *p* < 0.001
Limits of agreement	166.8 (−1177.8 to 1511.4)	−52.0 (−795.9 to 689.9)	−139.0 (−700.9 to 422.8)

Dice coefficient presented as mean and (SD)

*ICC* intraclass correlation coefficient

### Paired comparisons of matched plaques in the intraobserver test dataset

**Fig. 5:**
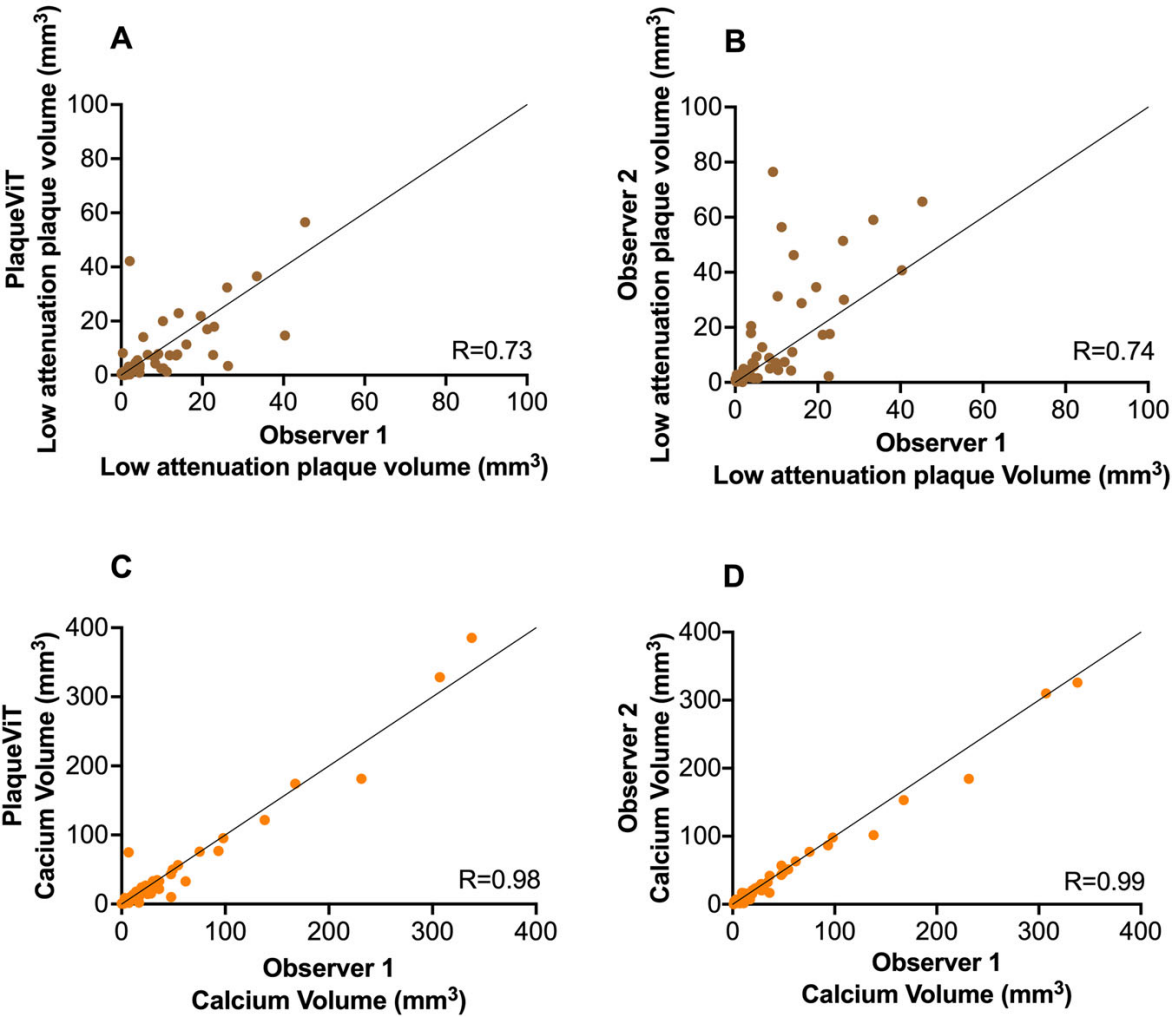
Correlations between segmented volumes plaque components in the interobserver variability dataset. Correlation plots of low-attenuation plaque volume between PlaqueViT and Observer 1 (**A**) and between Observer 1 and Observer 2 (**B**). Correlation plots of calcium volume between PlaqueViT and Observer 1 (**C**) and between Observer 1 and Observer 2 (**D**)

To ensure a correct comparison of plaques we used pairwise analysis. The mean Dice coefficient for segmentation of paired plaques between PlaqueViT and expert was 0.56 in the intraobserver test dataset compared to 0.61 between Observer 1 and Observer 2. The median average surface distance between PlaqueViT segmentation and expert annotation was 0.30 mm in the intraobserver test dataset compared to 0.34 mm for Observer 1 versus Observer 2. Agreement was good for low-attenuation plaque volume (ICC = 0.73, *p* < 0.001) and excellent for plaque volume (ICC = 0.95, *p* < 0.001) and calcified plaque volume (ICC = 0.98, *p* < 0.001) (Fig. 5). Similarity metrics for the comparison of matched plaques are presented in Table 3.

**Table 3:** PlaqueViT model performance in plaque segmentation of matched plaques

Variable	PlaqueViT versus Observer 1 (*n* = 73)	Observer 1 versus Observer 2 (*n* = 69)
Segmentation of coronary plaques		
Dice coefficient	0.56 (0.16)	0.61 (0.17)
Median average surface distance (mm)	0.30	0.34
Plaque volume (mm^3^)		
Correlation	0.95, *p* < 0.001	0.96, *p* < 0.001
ICC	0.94, *p* < 0.001	0.95, *p* < 0.001
Limits of agreement	0.45 (−86.1 to 87.1)	10.3 (−69.6 to 90.3)
Mean absolute percentage error	41.6%	39.5%
Low-attenuation plaque volume (mm^3^)		
Correlation	0.73, *p* < 0.001	0.74, *p* < 0.001
ICC	0.73, *p* < 0.001	0.62, *p* < 0.001
Limits of agreement	−0.61 (−13.8 to 13.9)	3.78 (−20.5 to 28.1)
Mean absolute percentage error	73.8%	57.3%
Calcified plaque volume (mm^3^)		
Correlation	0.98, *p* < 0.001	0.99, *p* < 0.001
ICC	0.98, *p* < 0.001	0.99, *p* < 0.001
Limits of agreement	−0.79 (−27.8 to 26.3)	−2.69 (−19.0 to 13.6)
Mean absolute percentage error	35.8%	35.6%
Fibrous-fatty plaque volume (mm^3^)		
Correlation	0.87, *p* < 0.001	0.92, *p* < 0.001
ICC	0.86, *p* < 0.001	0.89, *p* < 0.001
Limits of agreement	1.30 (−31.6 to 34.2)	5.32 (−27.0 to 37.7)
Mean absolute percentage error	51.7% (46.1)	46.3% (36.1)
Fibrous plaque volume (mm^3^)		
Correlation	0.94, *p* < 0.001	0.98, *p* < 0.001
ICC	0.94, *p* < 0.001	0.98, *p* < 0.001
Limits of agreement	1.26 (−21.1 to 23.6)	24.4 (36.4 to 85.2)
Mean absolute percentage error	41.7% (36.2)	35.7% (33.7)

Similarity metrics of paired comparisons of all matched plaques in the Intraobserver test dataset. Data presented as correlation coefficient, intraclass correlation (ICC), limits of agreement (1.96 SD interval) or mean percentage error (standard deviation). Dice coefficient presented as mean and (SD). Similarity metrics for plaque burden and the remodeling index are shown in the supplement

### Model performance in CAD detection

The ability of PlaqueViT to detect any plaque > 2 mm^3^ in the intraobserver test dataset (*n* = 65) is shown in Table 4, along with corresponding intraobserver performance. The readings of Observer 1 were defined as ground truth. In the CAD detection dataset (*n* = 684), PlaqueViT exhibited a per-patient sensitivity of 0.95 and specificity of 0.83 for detecting any CAD. Table 4 shows the diagnostic performance of PlaqueViT.

**Table 4:** CAD detection

	Intraobserver test dataset (*n* = 65)		CAD detection dataset (*n* = 684)	External test dataset (*n* = 28)	
	PlaqueViT versus Observer 1	Observer 1 versus Observer 2	PlaqueViT versus radiologic assessment	PlaqueViT versus Observer 1	Expert reader versus Observer 1
Sensitivity	0.97	0.94	0.95	0.88	0.76
Specificity	0.76	0.86	0.83	0.91	1.0
Kappa	0.75	0.81	0.78	0.78	0.72
Agreement	0.88	0.91	0.89	0.89	0.86
Predictive value					
Positive	0.83	0.89	0.84	0.94	1.0
Negative	0.96	0.93	0.95	0.83	0.73
ICC	0.98	0.96	*	0.99	0.92

*CAD* coronary artery disease, *ICC* intraclass correlation coefficient

* Radiologic assessment had no volume data and ICC cannot be calculated

### Model performance in plaque detection in the external test dataset

The ability of PlaqueViT to detect any plaque > 2 mm^3^ in the external test dataset (*n* = 28) is reported in Table 4, along with the corresponding intraobserver performance. The Dice coefficient for total plaque volume was 0.51 for PlaqueViT versus Observer 1 and 0.37 for Observer 1 versus Observer 2. Performance of plaque segmentation in the pairwise analysis of matched plaques is reported in the supplement.

## Discussion

This study shows that our deep learning model, PlaqueViT, can rapidly detect and segment coronary artery plaques without manual pre- or postprocessing and does so as well as an expert reader. Segmentation by PlaqueViT is performed in a voxelwise fashion. Thus, it can quantify plaque features, including the volume of low-attenuation plaque tissue, which predicts the risk for myocardial infarction [4, 7]. Voxelwise segmentation will also make it possible to apply radiomic analysis methods to CCTA data [19] and identify specific structural features of coronary plaques that predict future myocardial events. In studies like SCAPIS, outcome data on myocardial infarcts can be used in future radiomic models to describe the types of coronary plaques that hold the highest risk.

Unlike other deep learning models for CCTA assessment, PlaqueViT does not require any pre- or postprocessing for coronary vessel segmentation. Previous models for voxelwise plaque segmentation typically rely on a three-step approach that involves semi-automatic centerline extraction, straightened or curved multiplanar reformation, and vessel contour delineation. The model of Lin et al [10] performs similarly to ours but requires manual centerline extraction. The model of Jávorszky et al [11] requires both manual centerline extraction and multiplanar reconstruction before the deep learning model is applied, and although it provides voxelwise plaque segmentation, ICC analysis shows weaker agreements than our model. Most previous models can only detect plaques, determine the CAD-RADS grade, and the degree of stenosis, or classify plaque type without providing actual plaque delineations. Like PlaqueViT, the model of Jin et al [12] is fully automatic and can detect, delineate and classify coronary plaques; however, the authors do not evaluate the voxelwise plaque segmentations. To the best of our knowledge, PlaqueViT is the first deep learning model that does not rely on any manual interventions to provide coronary plaque detection and voxelwise segmentation.

The PlaqueViT model is somewhat oversensitive, as judged by comparison to the visual assessments in SCAPIS, which could be due to a possible preselection bias from only including scans with plaques present in the training data. However, after importing the PlaqueViT segmentations into the QAngio CT workstation, we visually reassessed the first 50 cases with the largest segmented plaques classified as false positives in the CAD detection dataset and found that 73% had a true coronary plaque. In the majority of these cases, the plaques were noncalcified and contained low-attenuation plaque tissue. This finding suggests that automated reading has significant advantages for large studies involving many different radiologists with different levels of experience. Since our model is fully automatic, accurate, and fast—with a mean analysis time of 25 s per case—it is well suited to analyze and segment coronary plaques in large studies such as SCAPIS.

### Limitations

Our study has limitations. First, the training data were exclusively from the SCAPIS cohort, whose scans were acquired using a single type of CT scanner. Although the performance of PlaqueViT on the external test dataset (with a different type of CT scanner) showed promising results, the model needs to be evaluated on a broader range of CT scanners before it can be introduced into a clinical environment. Second, PlaqueViT was trained from annotations from one senior expert, whose reading was not verified against intravascular ultrasound data or event data. Third, PlaqueViT has not been trained or evaluated on CCTA scans where the overall image quality was considered too poor for manual plaque analysis by the expert reader. Finally, PlaqueViT has not been evaluated on stenosis classification using CAD-RADS classes or plaque classification (calcified/noncalcified/mixed).

## Conclusion

We developed PlaqueViT, a deep learning model for fully automatic plaque detection and segmentation that detects and delineates coronary plaques and the arterial lumen with similar performance as an experienced radiologist. PlaqueViT could be integrated into clinical practice, serving as both a supplementary reader and a valuable tool to support clinical decision-making. Because a model such as PlaqueViT can rapidly deliver automatic and unbiased results, it has the potential to reduce intraobserver variability and minimize interpretation errors made by physicians. It could be used for pre-screening to identify patients with coronary artery diseases and prioritize their CCTA scans for review by a radiologist.

## Supplementary information

Electronic Supplementary Material

## Data Availability

The authors do not have permission to share images. SCAPIS data and images can be shared from scapis.org. The trained model is the intellectual property of authors J.A., R.P., G.B., E.F. and O.H.

## Abbreviations

CAD: Coronary artery disease
CCTA: Coronary computed tomography angiography
CT: Computed tomography
ICC: Intraclass correlation coefficient
SCAPIS: Swedish CardioPulmonary BioImage Study
