# Supplementary material for: PlaqueViT: a vision transformer model for fully automatic vessel and plaque segmentation in coronary computed tomography angiography
Source: Eur Radiol. 2025 Feb 5;35(8):4461–71. doi: 10.1007/s00330-025-11410-w (PMC12226657; doi:10.1007/s00330-025-11410-w)
Supplement: Supplementary file 1 — Electronic Supplementary Material [file 330_2025_11410_MOESM1_ESM.pdf]

PlaqueViT: A Vision Transformer Model for Fully Automatic Vessel and Plaque Segmentation in Coronary Computed Tomography Angiography

ELECTRONIC SUPPLEMENTARY MATERIAL

Definition of Measures

Plaque components. Plaque components were defined by their radiodensity in Hounsfield units (HU) as follows: low-attenuation plaque, -30–30 HU; fibrous fatty tissue, 31–150 HU; fibrous tissue, 151–350 HU; and dense calcium, 351–2048 HU.[1-3]

Plaque burden. Plaque burden at a particular slice was defined as the plaque area divided by the vessel area (lumen plus plaque area) at that location. Mean plaque burden was defined as the mean over all slices of the plaque.

Model development

Preprocessing and training. To prepare the input images, we applied preprocessing steps including resampling of the images to an isotropic voxel size of 0.33 x 0.33 x 0.33 mm^3, clipping voxel values to the range of [-1000, 1500] HU, and scaling voxel values to the range of [-1, 1]. The model analyzes the CCTA in a patch wise manner, with each image patch size set to 192 x 192 x 192 voxels. Thus, the field-of-view equals 63.36 x 63.36 x 63.36 mm^3, which is the largest cubic field-of-view the dataset allows for. Python version 3.8, PyTorch version 1.11.0, NumPy version 1.24.4, and SciPy version 1.10.1 were used for data preprocessing and model training.

Training settings for each ensemble member. The batch size was 5 samples per GPU. Training was done on sub volumes of size [192, 192, 192] voxels that were randomly sampled with higher weighting at foregrounds. AdamW[4] was used as an

optimizer; weight decay was set to 0.005. The learning rate was initialized at 0.001 and followed a cosine decay schedule with 2000 linear warmup iterations and a maximum of 80,000 iterations. Each model was initialized with PyTorch's default random weight initialization. To stabilize the training, gradients were clipped to have a maximum norm of 1.0. The Sharpness-aware minimization (SAM) with ρ=0.05 was also used during training[5]. For the loss function, we use a weighted average Dice loss with class weights of 0.1, 0.4, 0.1, 0.4 for the background, lumen, ostium, and plaque classes, respectively.

Ablation study. The different components of the nnFormer architecture were evaluated by comparing the mean Dice coefficient for plaque segmentations in one of the validation datasets, and the best setup was chosen, see the table below.

Residual Connection	Skip Attention	Deep Supervision	Stochastic Depth: 0.1	Global Attention Bottleneck	Local Attention Bottleneck	Mean Validation Plaque Dice
	✓	✓	✓	✓		0.43
✓		✓	✓	✓		0.47
✓			✓	✓		0.48
	✓		✓	✓		0.48
	✓				✓	0.47
**✓**					**✓**	**0.49**
✓				✓		0.48
✓		✓			✓	0.43
	✓	✓			✓	0.38
	✓		✓		✓	0.47

Table of ablation study. The mean Dice coefficient for segmented plaques in one of the validation datasets is presented for different model configurations from the nnFormer architecture. The best configuration is highlighted in bold.

Evaluation. —Straightened multiplanar reconstruction (MPR) images were used to match the location of plaques in the matched analysis. MPR images were generated with a custom Python script and 3D Slicer's Python API[6] with the extension

packages SlicerVMTK and Sandbox. The following steps were applied. First, a straightened MPR image was generated for each segmented vessel branch by extracting an anchor point and end point. The anchor point was defined as the end point with the closest Euclidean distance to the center of the ostium in connection with the vessel branch, and the end point was located at the end of the vessel branch. If a vessel branch was missing a segmented ostium, its anchor point was defined as the end point with nearest Euclidean distance to the center of the corresponding volume. Second, the corresponding centerline was then generated by using its corresponding anchor point and end point. Third, the centerlines were subsequently used to generate the straightened MPR images.

Supplementary Results
Paired comparisons of all matched plaques in the intraobserver test dataset

		Model vs observer 1<br>N=73	Observer 1 vs observer 2<br>N=69
Plaque burden			
	Correlation	0.82 p<0.001	0.87, p<0.001
	ICC	0.82 p<0.001	0.87, p<0.001
	Limits of agreement	0.017 (0.139)	-0.027 (0.12)
Fibrous-fatty plaque volume			
	Correlation	0.87, p<0.001	0.92, p<0.001
	ICC	0.86, p<0.001	0.89, p<0.001
	Limits of agreement	1.30 (16.8)	5.32 (16.5)
	Mean Absolute Percentage Error	51.7% (46.1)	46.3% (36.1)
Fibrous plaque volume			
	Correlation	0.94, p<0.001	0.98, p<0.001
	ICC	0.94, p<0.001	0.98, p<0.001
	Limits of agreement	1.26 (11.4)	24.4 (31)
	Mean Absolute Percentage Error	41.7% (36.2)	35.7% (33.7)

Paired comparisons of all matched plaques in the external test dataset

		Model vs observer 1<br>N=41	External reader vs observer 1<br>N=37
Segmentation of coronary Plaques			
	Dice coefficient	0.62 (0.11)	0.57 (0.18)
	Median average surface distance (mm)	0.23	0.35
Plaque Volume			
	Correlation	0.98 p<0.001	0.91, p<0.001
	Limits of agreement	12.5 (31.1)	-24.2 (49.3)
	Mean % difference	-3.40% (38.2)	-38.8% (48.8)
	Mean Absolute % difference	27.7% (38.2)	49.1% (38.0)
Low attenuation plaque volume			
	Correlation	0.83, p<0.001	0.81, p<0.001
	Limits of agreement	-0.18 (4.06)	-1.10 (3.87)
	Mean % difference	-18.5% (69.7)	38.7% (63.6)
	Mean Absolute % difference	52.0% (49.4)	56.3% (48.1)

Dice coefficient presented as mean and (SD).

References

1 Motoyama S, Sarai M, Harigaya H et al (2009) Computed tomographic angiography characteristics of atherosclerotic plaques subsequently resulting in acute coronary syndrome. J Am Coll Cardiol 54:49-57

2 de Graaf MA, Broersen A, Kitslaar PH et al (2013) Automatic quantification and characterization of coronary atherosclerosis with computed tomography coronary angiography: cross-correlation with intravascular ultrasound virtual histology. Int J Cardiovasc Imaging 29:1177-1190

3 Brodoefel H, Reimann A, Heuschmid M et al (2008) Characterization of coronary atherosclerosis by dual-source computed tomography and HU-based color mapping: a pilot study. Eur Radiol 18:2466-2474

4 Loshchilov I, Hutter F (2017) Decoupled Weight Decay Regularization.

5 Foret P, Kleiner A, Mobahi H, Neyshabur B (2020) Sharpness-aware minimization for efficiently improving generalization. arXiv preprint arXiv:201001412

6 Available via https://slicer.readthedocs.io/en/latest/developer_guide/api.html#python
